# Leisure-Time Physical Activity Trajectories across Adulthood and Cardiometabolic Risk at the Beginning of Late Adulthood: A Prospective Cohort Study

**DOI:** 10.1249/MSS.0000000000003883

**Published:** 2025-10-27

**Authors:** TIINA SAVIKANGAS, KATJA KOKKO, JOHANNA AHOLA, TIIA KEKÄLÄINEN, MARJA-LIISA KINNUNEN, EMMI REINILÄ, EERO A. HAAPALA

**Affiliations:** 1 Gerontology Research Center and Faculty of Sport and Health Sciences, University of Jyväskylä, Jyväskylä, FINLAND; 2 Laurea University of Applied Sciences, Vantaa, FINLAND; 3 The Wellbeing Services County of Central Finland, Jyväskylä, FINLAND; 4 School of Medicine, University of Eastern Finland, Kuopio, FINLAND; 5 Sports & Exercise Medicine, Faculty of Sport and Health Sciences, University of Jyväskylä, Jyväskylä, FINLAND; 6 Institute of Biomedicine, School of Medicine, University of Eastern Finland, Kuopio, FINLAND; 7 Children’s Health and Exercise Research Centre, Faculty of Health and Life Sciences, University of Exeter, Exeter, UNITED KINGDOM

**Keywords:** BLOOD PRESSURE, CENTRAL OBESITY, DYSLIPIDAEMIA, METABOLIC SYNDROME, MIDLIFE, TRAJECTORY

## Abstract

**Purpose:**

Physical activity (PA) is a key strategy in preventing and treating metabolic syndrome (MetS). The purpose is to investigate the associations of leisure-time PA (LTPA) trajectories across adulthood and current PA with MetS at age 61.

**Methods:**

Participants were 159 Finnish adults (52% women). LTPA frequency was assessed at ages 27, 42, 50, and 61 with a single question. Current PA at age 61 included self-reported vigorous, muscle-strengthening, commuting, and occupational PA. Cardiometabolic risk factors at age 61 included waist circumference, blood pressure, high-density lipoprotein cholesterol, triglycerides, and glucose. MetS was defined based on the Adult Treatment Panel III criteria. LTPA trajectories were conducted using k-means for longitudinal data.

**Results:**

Of the three LTPA trajectories found, consistently inactive (*N* = 34) and increasingly active (*N* = 58) had a higher risk of MetS compared with consistently active (*N* = 67) (odds ratio [95% confidence interval]: 3.93 [1.55–10.01] and 2.39 [1.14–4.99], respectively). Only the difference between consistently inactive and consistently active remained statistically significant when the current PA indicators were included in the model. Considering the individual components of MetS, those who were consistently inactive and increasingly active had higher waist circumference, lower high-density lipoprotein, and higher triglyceride levels compared with consistently active. These differences did not remain statistically significant when current PA was included.

**Conclusions:**

Although consistently inactive and increasingly active individuals had higher cardiometabolic risk at age 61 compared with those who were consistently active across adulthood, current PA participation at the beginning of late adulthood attenuated these risks. These findings emphasize the importance of promoting and sustaining PA throughout life to reduce the burden of MetS in the aging population.

Physical inactivity remains a significant public health concern (1), and the prevalence of insufficient physical activity (PA) continues to rise (2). Moreover, the prevalence of overweight and obesity (3), central obesity (4), hypertension (5), and impaired glucose metabolism (6) has increased during the last 2 decades. When these cardiometabolic risk factors, along with dyslipidemia, are clustered together, the condition is referred to as metabolic syndrome (MetS). MetS increases the risk of cardiometabolic diseases, such as heart failure (7), coronary heart disease, and type 2 diabetes mellitus (8), but also elevates the risk of vascular dementia (9), mental health problems (10), and musculoskeletal disorders (11). Therefore, preventing and managing MetS has become a critical public health priority, with significant implications for individual well-being and healthcare costs.

PA has been considered one of the most important modifiable therapeutic strategies in preventing and treating MetS and its components (12,13). PA is defined as any bodily movement produced by skeletal muscles that results in energy expenditure, while exercise is planned, structured, and repetitive PA, which is designed to improve or maintain physical fitness (14). The evidence from randomized controlled trials focuses on the effects of exercise training and suggests that exercise is effective in preventing and treating MetS (15–17). However, randomized controlled trials are usually relatively short (≤12 months) and do not consider the continuation of PA after the intervention, and may, thus, not reflect the effects of lifelong exposure to PA on MetS. In the prevention of MetS over the lifespan, the different domains of PA may provide divergent benefits on cardiometabolic health and explain the associations between total PA and cardiometabolic risk factors. For example, evidence is emerging that while leisure-time PA (LTPA) and active commuting are beneficial for cardiometabolic health, occupational PA may even be associated with adverse cardiometabolic health outcomes (18–21). Moreover, the beneficial associations between total PA and cardiometabolic risk factors may be driven by LTPA (18).

Few studies have investigated the role of PA trajectories in health outcomes over several decades. A recent study based on a Finnish Twin Cohort Study found that individuals who remained continuously physically inactive (low PA trajectory) over a 36-yr follow-up from young adulthood to the onset of late adulthood had higher self-reported body mass index (BMI) and a higher prevalence of self-reported hypertension and type 2 diabetes mellitus than other PA trajectories (22). Similarly, a low and decreasing PA trajectory over 20 yr was associated with a higher risk of all-cause and cardiovascular mortality and cardiovascular events in individuals who were 40–59 yr at the baseline (23). In a shorter follow-up of 6 yr, including adults aged 50 yr and older, individuals with a low PA trajectory had an increased risk of type 2 diabetes mellitus (24). Moreover, some evidence suggests similar associations in adolescents and young adults, with those with a low PA and decreased PA trajectories having a high BMI and impaired glucose metabolism (25,26). However, there is limited evidence on the associations of long-term PA trajectories from young adulthood with late adulthood MetS and its components assessed using laboratory-based biomarkers.

Another gap in the existing literature is that research is lacking on whether current PA participation contributes to the relationship between long-term PA exposure and the development of MetS. The current PA guidelines suggest that any PA is beneficial for health, but different PA modalities, including different types (e.g., aerobic and muscle-strengthening PA) and domains (e.g., leisure-time, occupational PA), have distinct effects on cardiometabolic outcomes (27). Furthermore, while it has been suggested that engaging in PA at any age provides cardiometabolic health benefits (27), little is known about whether consistently engaging in high levels of PA (high PA trajectory) provides additional benefits over more recent PA participation. Classical models of life course epidemiology of chronic diseases suggest that timing, context, and accumulation of as well as change in a behavior may determine its health risks (28), highlighting the need to investigate both longitudinal and current PA behavior jointly.

Therefore, the aims of the current study were to 1) identify LTPA trajectories from young adulthood to the beginning of late adulthood, 2) investigate the associations of identified LTPA trajectories with MetS and whether these associations remain after taking the current PA participation into account, 3) investigate the associations of identified LTPA trajectories with MetS components and whether these associations remain after taking the current PA participation into account.

## METHODS

### Study Design and Participants

This study uses data from the ongoing Jyväskylä Longitudinal Study of Personality and Social Development (JYLS) (29), especially its latest data collection phase TRAILS (Developmental Psychological Perspectives on Transitions at Age 60: Individuals Navigating Across the Lifespan) (30), conducted at the University of Jyväskylä, Finland. Initially, twelve complete primary school second-grade classes from the city center areas and suburban areas in Jyväskylä, Central Finland, were randomly selected, and all children in those classes (*N* = 369, 53% males) were included in the JYLS study in 1968 when the children were approximately 8 yr old (29). Thereafter, major data collections were conducted at the ages of 14, 27, 36, 42, 50, and 61. The participants were surveyed for the first time on their LTPA at the age of 27 yr, and the present study is based on data collection waves in 1986, 2001, 2009, and 2020–2021, when the participants were approximately 27, 42, 50, and 61 yr old, respectively. These data collection points were chosen since they included comparable data on LTPA.

The latest data collection, TRAILS, was conducted between February 2020 and July 2021 when the participants were about 61 yr old. The data collection has been described in detail by Kokko et al. (30). Briefly, all eligible participants who had not withdrawn from the study and whose contact information was available (*N* = 301) were mailed an information letter, a written informed consent form, and a Life Situation Questionnaire (LSQ) on February 11, 2020. Participants were invited to attend psychological interviews and health examinations. Data collection was completed on July 1, 2021. The participants who remained in the study at age 61 (*N* = 206) were still relatively well representative of the age cohort born in 1959 in Finland (30). The present study includes the participants (*N* = 159) who completed the LSQ and health examination during the latest data collection wave and thus had valid data on LTPA and cardiometabolic risk at age 61.

### Measures

#### Physical Activity

##### Leisure-time physical activity

General LTPA level was self-reported using a single-item question presented in the LSQ at the ages of 27, 42, 50, and 61 yr (31,32). At the age of 27, the question was “How often do you do physical exercise or sport?” and had the following five response options: 1 = not at all, 2 = less than once a week, 3 = about once a week, 4 = 2–4 times/week, 5 = almost daily. During the three later data collection waves, the question had a slightly more detailed phrasing and read as follows: “How often do you exercise (including incidental exercise) or pursue sports in your leisure time?.” The question had the following seven response options: 1 = never, 2 = less than once a month, 3 = 1–2 times/month, 4 = once a week, 5 = 2–3 times/week, 6 = 4–5 times/week, 7 = practically every day. The response categories 2 and 3, as well as categories 5 and 6, were combined for the trajectory analysis to match the response options in the questionnaire at the age of 27 as well as possible. Thus, the final LTPA categories used in the trajectory analysis from the age of 42 to 61 were as follows: 1 = never, 2 = twice per month or less frequently, 3 = once a week, 4 = 2–5 times/week, 5 = practically every day.

During the latest data collection wave at the age of 61 yr, more detailed information about current participation in different PA modalities, including muscle-strengthening PA, vigorous-intensity PA, commuting PA, and occupational PA, was collected. Participants were asked about their engagement in vigorous-intensity PA in the LSQ and about other PA modalities in the psychological interview. The current PA participation was included in the present analysis to provide insights into the importance of various PA modalities on cardiometabolic risk factors at the onset of late adulthood.

##### Vigorous PA

The following question was used to assess engagement in vigorous-intensity PA: “How often do you exercise or do sports in your free time for at least half an hour, being out of breath and sweating?” (33). The seven-point response scale was like in the general LTPA question and was recategorized as participating in vigorous PA every week (categories 3–6) vs. less frequently (categories 0–2).

##### Muscle-strengthening PA

Weekly engagement in muscle-strengthening PA was assessed with the following two questions: “In your leisure time, do you regularly engage in muscle-strengthening physical activity (e.g., circuit training or gym training)?” (yes vs. no), and “If yes, how many times per week?” (34). Participants who responded “yes” to the first question and one or more times per week to the second question were categorized as engaging in weekly muscle-strengthening PA, and others as not engaging in weekly muscle-strengthening PA.

##### Commuting PA

The following question was used to assess daily work-related commuting PA: “How much of your daily journey to work is spent in walking, cycling and/or running? (according to the most typical journey).” Response scale was 1 = I am presently not at work; 2 = No physical activity on journeys to work; 3 = 1–15 min; 4 = 15 min to less than half an hour; 5 = half an hour to less than an hour; 6 = an hour or more (35). Participants selecting options 3–6 were categorized as regularly engaging in commuting PA, whereas participants selecting categories 1 and 2 were categorized as not engaging in commuting PA.

##### Occupational PA

Finally, participation in occupational PA was assessed with the following question: “What type is your current occupation?” The response options were 1 = not working, 2 = mostly sedentary activity, 3 = mostly standing and walking, 4 = lifting and carrying in addition to standing and walking, and 5 = physically heavy work (35). Engagement in occupational PA was categorized as yes (categories 3–5) vs. no (categories 1–2).

#### Cardiometabolic risk factors

The five cardiometabolic risk factors determining MetS at age 61 included waist circumference, high-density lipoprotein (HDL) cholesterol, triglycerides, blood glucose, and blood pressure. Waist circumference and blood pressure were assessed during the health examination by the study nurse, while the blood samples were taken either at the laboratory of the University of Jyväskylä or at local health centers. The samples taken elsewhere were sent to and analyzed at the university laboratory (30). Waist circumference was measured midway between the lowest rib margin and the iliac crest to the nearest 1 cm. Fasting plasma HDL cholesterol, triglycerides, and glucose levels (mmol/L) were determined by enzymatic methods using automatic analyzer equipment (Konelab, Vantaa, Finland) from venous blood samples drawn after a 12-h overnight fast. Resting systolic and diastolic blood pressure (mm Hg) were measured twice using a standard automatic sphygmomanometer in a sitting position after 15 min of rest. Measurements were taken from the right arm to the nearest 2 mm Hg. The mean values of the two measurements were calculated and used as the outcomes.

##### Prevalence of MetS

The participants were classified as having a particular risk factor based on the following thresholds according to the Adult Treatment Panel III criterion (35): waist circumference >88 cm for women and >102 cm for men, HDL cholesterol <1.29 mmol/l for women and <1.04 mmol/l for men, triglycerides ≥1.7 mmol/l, plasma glucose ≥6.1 mmol/l, and systolic blood pressure ≥130 and/or diastolic blood pressure ≥85 mm Hg. If the participants had dyslipidemia, diabetes, and/or blood pressure medication at age 61, or had previously reported using these medications, they were classified as having that risk factor, even if the values were within thresholds (36). Medication was self-reported in the nurse’s examination and coded according to the ATC classification. Participants were coded as users of dyslipidemia, diabetes, and/or blood pressure medication if they had at least one medication for the respective condition in regular use. The prevalence of MetS was categorized as no (0–2 risk factors) versus yes (3–5 risk factors). The Adult Treatment Panel III criteria were selected since they have been used in previous phases of the JYLS study (37,38).

#### Descriptive characteristics

Gender was defined as male or female during the first data collection wave of JYLS in 1968. Body weight (kg) was measured with a digital scale and height (cm) with a wall-mounted stadiometer by the study nurse during the health examination, and BMI (kg/m^2^) was calculated.

The health-related and socioeconomic characteristics of the participants were drawn from the LSQ and included self-reported health (very good/good vs average/poor/very poor), occupational status based on job title (upper white-collar vs. lower white-collar vs. blue-collar worker), and current employment status (in employment vs. not in employment). Employment status was originally reported as being 1) in full-time employment, 2) in part-time employment, 3) entrepreneur, 4) unemployed, 5) laid off, 6) retired, or 7) job alternation leave. Categories 1–3 were classified as “in employment” and categories 4–7 as “not in employment.”

### Statistical Analysis

To form longitudinal LTPA clusters, that is, LTPA trajectories across adulthood, an analysis was performed using a k-means algorithm for longitudinal data (KmL) (39). KmL was chosen since it is suggested to be effective in relatively small samples with few data points (40). Furthermore, KmL benefits from not expecting a specific (e.g., normal) distribution of the outcome variable or a specific shape of subgroup trajectories but allows different subgroups to have different trajectory shapes (e.g., linearly increasing, U-shaped).

The KmL method grouped observations of LTPA at the four measurement points (ages 27, 42, 50, and 61) into homogeneous subgroups, that is, clusters that were as heterogeneous as possible from each other. The KmL method assigns each observation to a cluster, and the optimal solution is reached by altering two phases: 1) determining the center of each cluster and 2) assigning each observation to its nearest cluster (39). We allowed the KmL to run for two to six clusters, 20 times each, to find the optimal number of clusters and the best solution. We run the KmL algorithm in the full JYLS study sample and set the algorithm to exclude all participants with missing LTPA data in more than two data collection waves. This approach resulted in 296 participants being included in the LTPA trajectory analysis. For them, the KmL algorithm was set to automatically impute missing data with the last onset carried forward method, that is, if a participant had missing LTPA information at a given time point, it was imputed with the LTPA category from the previous time point with existing data. The first criterion to select the final solution was the Calinski and Harabatz criterion, that is, the optimal number of clusters is the number that maximizes the between-matrix variance and minimizes the within-matrix variance (39). Based on this Calinski-Harabatz score, the best solutions for two and three clusters were almost equally good. The decision was thus also based on the potentially clinically meaningful information provided by the three-cluster model in comparison to the two-cluster model, that is, three LTPA trajectories were identified in the KmL analysis. This is in line with the common practice of validating cluster selection by expert opinion (39,41).

Participant characteristics at age 61 are presented as means and standard deviations for continuous variables and as frequencies and percentages (%) for categorical variables in the full analytical study sample (*N* = 159) and according to gender. Differences across LTPA trajectories in current participation at age 61 in the different PA modalities (i.e., vigorous, muscle-strengthening, commuting, and occupational PA) were analyzed with Pearson’s Chi-squared tests.

Binary logistic regression analyses were performed to assess the associations of LTPA trajectories and current participation in different PA modalities with the prevalence of MetS at age 61. Only the LTPA trajectory was included as the predictor in the initial model, adjusted for gender (Model 1). Second, to investigate the independent associations of LTPA trajectories across adulthood and current PA with the prevalence of MetS, the four variables representing participation in different PA modalities at age 61 were added (Model 2). Results are presented as odds ratios (OR) and their 95% confidence intervals (CIs).

General linear models and generalized linear models with gamma link function were used to assess the associations between PA and the level of individual cardiometabolic risk factors. Each outcome, that is, waist circumference, HDL cholesterol, triglycerides, glucose, systolic and diastolic blood pressure, was analyzed separately. Initial models included the LTPA trajectory as the predictor, and the models were adjusted for gender and medication affecting the outcome variable in question (Model 1). Next, the four variables representing participation in different PA modalities, that is, current PA at age 61, were added (Model 2). Results are presented as unstandardized regression estimates (B), standard errors, and 95% CIs.

In all models, complete case analyses were performed. The main reasons for missing data were not consenting to the assessment in question, not attending the on-site health examination, or the interview. We imputed missing data for height for four participants from a previous data collection wave. We did not impute missing data in outcome or predictor variables since significant changes can occur in cardiometabolic outcomes over 11 yr, and information on participation in various PA modalities was not collected during the previous waves. Assumptions of regression models were checked based on the variance inflation factor, Shapiro-Wilk tests, and visual inspection of predictor value and residual histograms, residual vs. predicted value scatterplots, and Q–Q plots. Initial inspection revealed a nonlinear distribution of triglyceride and glucose values and their residuals in general linear models, indicating right-skewed distributions. Therefore, the associations of LTPA trajectories and current participation in different PA modalities with triglyceride and glucose levels were assessed with generalized linear models with gamma link function, which resulted in a better model fit based on Akaike’s information criterion values. For other outcomes, results from linear models are reported, since all assumptions were met.

The KmL algorithm was conducted, and the LTPA trajectories were formed in the R programming environment (version 4.3.1) with the package KmL (42). Other statistical analyses were conducted in jamovi software, version 2.2.5 (43). Generalized linear models were conducted using the module GAMLj (44).

## RESULTS

### Participants

**FIGURE 1.**
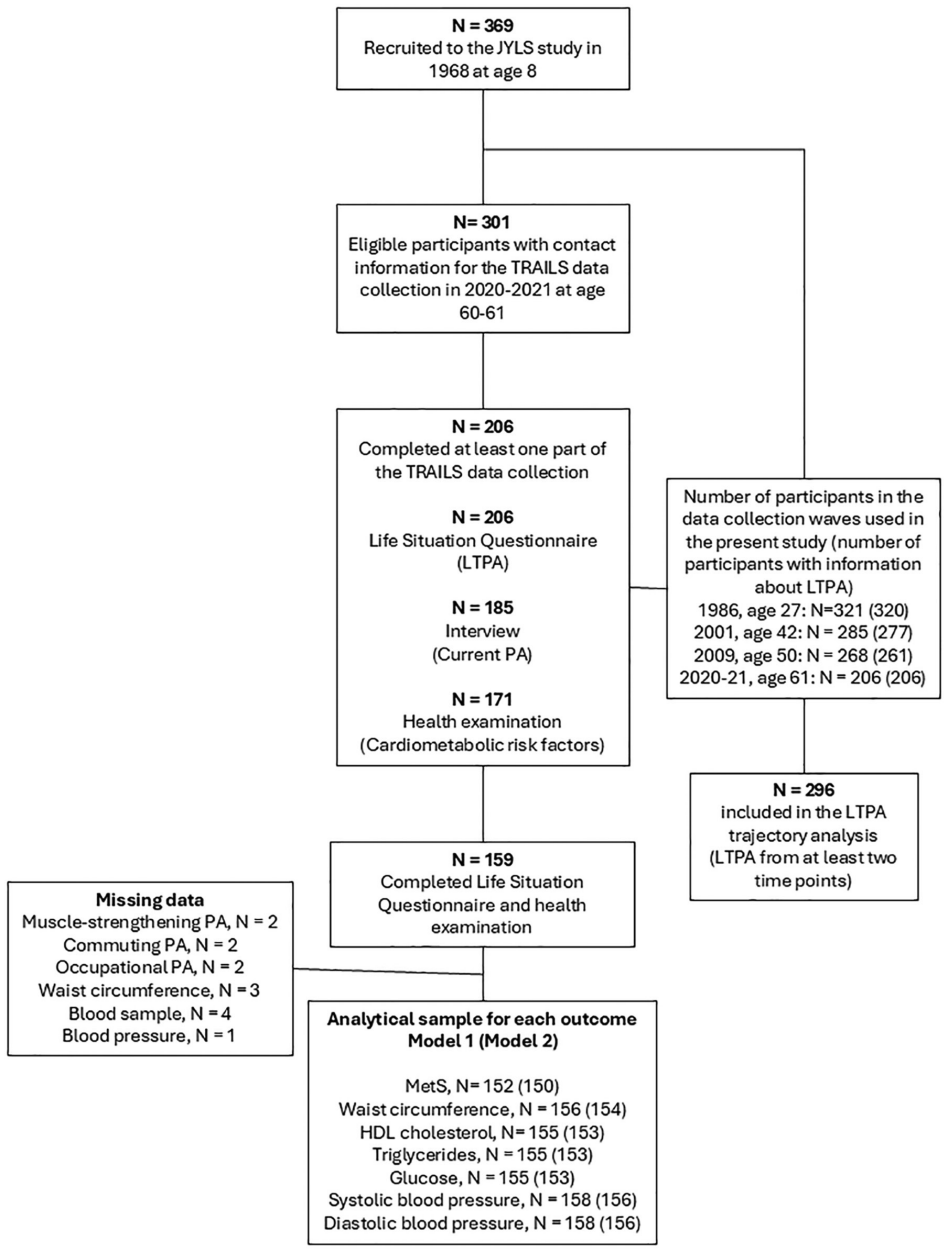
Study flow and the number of participants analyzed for each outcome and model.

The analytical sample of the present study included the participants (*N* = 159) who were included in the LTPA trajectory analysis and, in addition, had valid data on current PA and cardiometabolic risk factors at age 61. Due to missing data, the number of complete cases included in each analysis varied from 152 to 158 according to outcome and model (Fig. 1). The analytical sample did not differ from those excluded in key characteristics (Supplemental Table 1, Supplemental Digital Content, https://links.lww.com/MSS/D315).

### Leisure-time Physical Activity Trajectories

**FIGURE 2.**
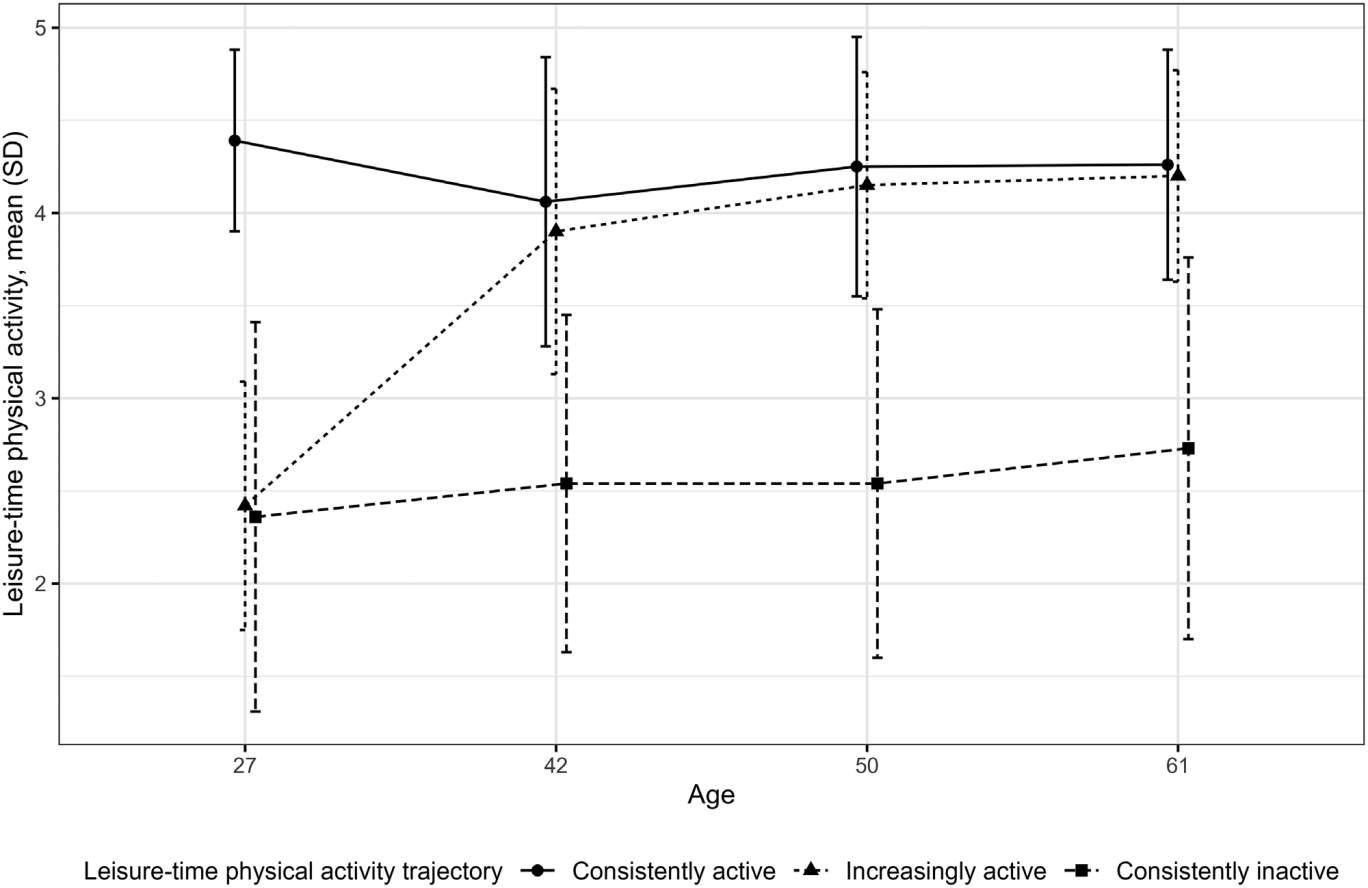
Leisure-time physical activity trajectories across adulthood in the Jyväskylä Longitudinal Study of Personality and Social Development (*N* = 296). Means and standard deviations (SD) of leisure-time physical activity frequency are presented by trajectory at each measurement time point. Leisure-time physical activity frequency at age 27: 1 = not at all, 2 = less than once a week, 3 = approximately once a week, 4 = 2–4 times/week, 5 = almost every day. Leisure-time physical activity frequency from the age 42 to 61: 1 = never, 2 = twice per month or less frequently, 3 = once a week, 4 = 2–5 times/week, 5 = practically every day.

In total, 296 participants were included in the KmL analysis. Three LTPA trajectories were identified and named based on the LTPA frequency: consistently active (40%), increasingly active (36%), and consistently inactive (24%) (Fig. 2). The distribution of participants across these three trajectories was similar in the analytical sample of the present study (*N* = 159). In the analytical sample, the median LTPA level of the consistently active participants (*N* = 67) was 4 across all measurements, that is, they typically engaged in LTPA several times per week. For increasingly active participants (*N* = 58), the median level was 3, that is, once a week, at age 27 and 4 in all subsequent measurements. The median LTPA level of consistently inactive participants (*N* = 34) was 2, that is, less than once a week, from the age of 27 to 42 and 3 (once a week) from the age of 50 to 61.

### Descriptive Characteristics

Participant characteristics are shown in Table 1 in the full analytical study sample and according to gender. Three of four participants engaged in vigorous PA and almost half of them in muscle-strengthening PA every week. Approximately four of ten participants engaged in regular commuting PA and were in physically active occupations. The number of cardiometabolic risk factors in the same individual varied from 0 to 5, and 51% of the participants had 3 or more risk factors, that is, they were categorized as having MetS.

**TABLE 1.** Participant characteristics in the whole study sample and by gender.

	All (*N* = 159)	Female (*N* = 83)	Male (*N* = 76)
Height, cm, mean ± SD	171.0 ± 8.6	164.9 ± 5.7	177.7 ± 5.7
Weight, kg, mean ± SD^a^	83.1 ± 17.5	75.4 ± 15.9	91.6 ± 15.1
BMI, kg/m^2^, mean ± SD^a^	28.4 ± 5.2	27.8 ± 5.6	29.0 ± 4.6
BMI ≥ 30 kg/m^2^, *n* (%)^a^	60 (38)	29 (35)	31 (41)
Good/very good self-reported health, *n* (%)	110 (69)	62 (75)	48 (63)
Currently in employment, *n* (%)	114 (72)	59 (71)	55 (72)
Occupational status, *n* (%)			
Upper white-collar	50 (31)	4 (5)	37 (49)
Lower white-collar	68 (43)	56 (67)	12 (16)
Blue-collar	41 (26)	23 (28)	27 (36)
Physical activity			
LTPA trajectory, *n* (%)			
Consistently active	67 (42)	39 (47)	28 (37)
Increasingly active	58 (36)	33 (40)	25 (33)
Consistently inactive	34 (21)	11 (13)	23 (30)
Weekly vigorous PA, yes, *n* (%)	116 (73)	61 (73)	55 (72)
Weekly muscle-strengthening PA, yes, *n* (%)^a^	72 (46)	42 (51)	30 (40)
Regular commuting PA, yes, *n* (%)^a^	66 (42)	40 (49)	26 (35)
Occupational PA, yes, *n* (%)^a^	59 (38)	29 (35)	30 (40)
Cardiometabolic risk factors			
Waist circumference, cm, mean ± SD^b^	97.8 ± 16.6	91.8 ± 15.5	103.6 ± 11.3
HDL cholesterol, mmol/L, mean ± SD^c^	1.58 ± 0.49	1.72 ± 0.53	1.44 ± 0.39
Triglycerides, mmol/L, mean ± SD^c^	1.53 ± 0.89	1.47 ± 0.70	1.61 ± 1.06
Glucose, mmol/L, mean ± SD^c^	5.83 ± 0.94	5.67 ± 0.77	6.01 ± 1.07
Systolic blood pressure, mm Hg, mean ± SD^d^	146.3 ± 18.9	143.7 ± 19.9	149.2 ± 17.3
Diastolic blood pressure, mm Hg, mean ± SD^d^	87.2 ± 10.0	85.1 ± 10.0	89.4 ± 9.6
Use of medication, *n* (%)			
Hypertension medication	62 (39)	27 (33)	35 (46)
Hyperlipidemia medication	36 (23)	14 (17)	22 (29)
Diabetes medication	14 (9)	5 (6)	9 (12)
Number of risk factors, *n* (%)^e^			
0–1	37 (23)	25 (30)	12 (16)
2	35 (23)	18 (22)	17 (24)
3	30 (20)	17 (21)	13 (18)
4	27 (18)	15 (19)	12 (17)
5	23 (15)	6 (7)	17 (24)
Prevalence of MetS, *n* (%)^e^	80 (53)	38 (47)	42 (59)

a Missing *N* = 2.

b Missing *N* = 3.

c Missing *N* = 4.

d Missing *N* = 1.

e Missing *N* = 7.

BMI, body mass index; HDL, high-density lipoprotein; LTPA, leisure-time physical activity; MetS, metabolic syndrome; PA, physical activity; SD, standard deviation.

Table 2 presents the associations between LTPA trajectories and participation in different PA modalities at age 61. Statistically significant between-group differences were observed in vigorous and muscle-strengthening PA (*P* < 0.001 for both), but not in commuting or occupational PA. Over 80% of consistently active and increasingly active, but less than 40% of consistently inactive participants engaged in vigorous PA weekly. Approximately 60%, 40%, and 20% of consistently active, increasingly active, and consistently inactive participants engaged in weekly muscle-strengthening PA, respectively. Overall, approximately 40% of participants had regular commuting PA and had physically active occupations.

**TABLE 2.** Number and proportion of participants in each leisure-time physical activity trajectory who engage in different physical activity modalities at age 61.

	Consistently Active (*N* = 67)	Increasingly Active (*N* = 58)	Consistently Inactive (*N* = 34)	*P* for Differences
Weekly vigorous PA, *n* (%)	55 (82)	48 (83)	13 (38)	<0.001
Weekly muscle-strengthening PA, *n* (%)	41 (61)	25 (44)	6 (18)	<0.001
Regular commuting PA, *n* (%)	33 (49)	20 (35)	13 (39)	0.265
Occupational PA, *n* (%)	30 (45)	20 (35)	9 (27)	0.210

### Associations of Leisure-Time Physical Activity Trajectories with the Prevalence of MetS

Compared with participants who were consistently active across adulthood, increasingly active and consistently inactive participants had 2.39 (95% CI: 1.14–4.99) and 3.93 (95% CI: 1.55–10.01) fold higher odds of having MetS at age 61 after adjustment for gender (Table 3, Model 1). When current participation in different PA modalities at age 61 was included in the model, only the higher risk perceived in consistently inactive participants compared with consistently active remained statistically significant (OR = 3.15, 95% CI: 1.08–9.13). In addition, not engaging in weekly muscle-strengthening PA had a borderline statistically significant association with higher odds of having MetS at age 61 compared with those who engaged in muscle-strengthening PA every week (OR = 2.12, 95% CI: 0.99–4.53). Participation in other PA modalities at age 61 was not associated with the prevalence of MetS (Table 3, Model 2).

**TABLE 3.** Associations of LTPA trajectories and current PA with the prevalence of MetS at age 61.

Model 1	Odds Ratio	95% CI Lower	95% CI Upper	*P*
Consistently active	Ref.			
Increasingly active	**2.39**	**1.14**	**4.99**	**0.021**
Consistently inactive	**3.93**	**1.55**	**10.01**	**0.004**
Male gender	1.43	0.73	2.81	0.299
Model				
Chi-squared	12.83			
Df	3			
*P* for model	0.005			
AIC	205.46			
Nagelkerke *R*^2^	0.11			
AUC	0.66			

Model 2				
Consistently active	Ref.			
Increasingly active	1.88	0.87	4.07	0.107
Consistently inactive	**3.15**	**1.08**	**9.13**	**0.035**
No weekly vigorous PA	0.62	0.26	1.48	0.280
No weekly muscle-strengthening PA	2.12	0.99	4.53	0.053
No regular commuting PA	1.69	0.83	3.42	0.146
No occupational PA	1.64	0.78	3.42	0.191
Male gender	1.30	0.64	2.64	0.470
Model				
Chi-squared	19.42			
Df	7			
*P* for model	0.007			
AIC	204.28			
Nagelkerke *R*^2^	0.16			
AUC	0.72			

AIC, Akaike’s information criterion; AUC, area under curve; CI, confidence interval; Df, degrees of freedom; LTPA, leisure-time physical activity; MetS, metabolic syndrome; PA, physical activity; Ref., reference category. Statistically significant associations are bolded.

### Associations of Leisure-Time Physical Activity Trajectories with Individual Cardiometabolic Risk Factors

Compared with consistently active participants, increasingly active and consistently inactive participants had greater waist circumference (B = 5.23, 95% CI: 0.49–9.96 and B = 9.18, 95% CI: 3.47–14.88, respectively), lower HDL cholesterol (B = −0.22, 95% CI: −0.38 to −0.05 and B = −0.22, 95% CI: −0.41 to −0.02, respectively), and higher triglyceride levels (B = 0.35, 95% CI: 0.07–0.64 and B = 0.51, 95% CI: 0.14–0.94), when the model only was adjusted for gender and relevant medication (Table 4, Model 1). Inspecting 95% CIs of estimated marginal means did not reveal statistically significant differences between increasingly active and consistently inactive participants in these outcomes.

**TABLE 4.** Associations of LTPA trajectories and PA at age 61 with cardiometabolic risk factors.

	Waist Circumference		HDL Cholesterol		Triglycerides		Glucose		Systolic Blood Pressure		Diastolic Blood Pressure	
	B (SE)	*P*	B (SE)	*P*	B (SE)	*P*	B (SE)	*P*	B (SE)	*P*	B (SE)	*P*
Model 1												
Intercept	88.50 (1.88)	<0.001	1.85 (0.86)	<0.001	1.52 (0.10)	<0.001	6.23 (0.17)	<0.001	145.19 (2.75)	<0.001	84.93 (1.43)	<0.001
Increasingly active	**5.23 (2.40**)	**0.031**	−**0.22 (0.08**)	**0.010**	**0.35 (0.14**)	**0.016**	0.16 (0.15)	0.277	−2.22 (3.42)	0.517	0.21 (1.78)	0.905
Consistently inactive	**9.18 (2.89**)	**0.002**	−**0.22 (0.10**)	**0.031**	**0.51 (0.19**)	**0.009**	0.09 (0.18)	0.631	−3.41 (4.12)	0.409	3.64 (2.14)	0.091
Male gender	10.57 (2.17)	<0.001	−0.24 (0.08)	0.002	−0.01 (0.13)	0.956	0.22 (0.13)	0.107	5.96 (3.10)	0.056	3.95 (1.61)	0.015
Medication			−0.14 (0.09)	0.115	0.29 (0.18)	0.097	1.41 (0.28)	<0.001	−0.36 (3.16)	0.910	−1.33 (1.64)	0.418
Model												
F	13.86		6.71		-		-		1.04		2.88	
Df	3		4		-		-		4		4	
Chi-squared/Df	-		-		0.28		0.02		-		-	
AIC	1255.16		202.57		319.16		368.65		1383.47		1176.70	
*R*^2^	0.21		0.15		0.12		0.23		0.03		0.07	

Model 2												
Intercept	82.67 (2.65)	<0.001	1.99 (0.09)	<0.001	0.96 (0.14)	<0.001	6.09 (0.23)	<0.001	142.36 (3.87)	<0.001	84.35 (2.05)	<0.001
Increasingly active	3.16 (2.43)	0.195	−0.16 (0.08)	0.064	0.21 (0.14)	0.142	0.12 (0.16)	0.424	−3.97 (3.51)	0.261	−0.32 (1.86)	0.863
Consistently inactive	6.19 (3.21)	0.056	−0.20 (0.11)	0.082	0.36 (0.21)	0.095	0.02 (0.20)	0.926	−5.10 (4.63)	0.272	3.45 (2.44)	0.160
No vigorous PA	−0.89 (2.65)	0.737	0.17 (0.09)	0.058	−0.10 (0.16)	0.527	−0.03 (0.17)	0.842	−0.11 (3.82)	0.977	−1.12 (2.02)	0.578
No muscle-strengthening PA	**5.11 (2.34**)	**0.031**	**−0.18 (0.08**)	**0.023**	0.25 (0.14)	0.064	0.11 (0.15)	0.473	4.37 (3.34)	0.193	1.64 (1.76)	0.354
No commuting PA	3.89 (2.19)	0.078	**−0.15 (0.07**)	**0.040**	0.007 (0.013)	0.598	0.11 (0.14)	0.450	5.18 (3.15)	0.102	1.26 (1.67)	0.450
No occupational PA	4.18 (2.23)	0.063	−0.06 (0.08)	0.469	0.25 (0.13)	0.052	0.02 (0.14)	0.868	−0.97 (3.24)	0.765	−0.40 (1.71)	0.814
Male gender	9.81 (2.19)	<0.01	−0.21 (0.08)	0.007	−0.03 (0.13)	0.823	0.21 (0.14)	0.134	4.73 (3.16)	0.137	3.57 (1.67)	0.034
Medication			−0.11 (0.09)	0.199	0.29 (0.17)	0.093	1.36 (0.29)	<0.001	−1.85 (3.23)	0.561	−1.68 (1.71)	0.328
Model												
F	7.49		4.84		-		-		1.07		1.23	
Df	7		8		-		-		8		8	
Chi-squared/Df	-		-		0.27		0.02		-		-	
AIC	1236.74		197.27		314.11		370.61		1368.27		11.69.32	
*R*^2^	0.26		0.21		0.15		0.22		0.005		0.008	

Results are reported from general linear models for waist circumference, HDL cholesterol, systolic and diastolic blood pressure, and from generalized linear models with gamma distribution for triglycerides and glucose. For LTPA trajectories, consistently active was set as the reference category. For all indicators of current PA, “yes” was set as the reference category.

AIC, Akaike’s information criterion; B, unstandardized regression coefficient; Df, degrees of freedom; HDL, high-density lipoprotein; LTPA, leisure-time physical activity; PA, physical activity; SE, standard error. Statistically significant associations are bolded.

When current participation in different PA modalities was included in the model, the between-group differences across LTPA trajectories in waist circumference, HDL, and triglycerides did not remain statistically significant (Table 4, Model 2). Not engaging in weekly muscle-strengthening PA at age 61 was statistically significantly associated with greater waist circumference (B = 5.1, 95% CI: 0.47–9.74) and lower HDL cholesterol (B = −0.18, 95% CI: −0.34 to −0.03). In addition, not performing regular commuting PA at age 61 was associated with lower HDL cholesterol (B = −0.15, 95% CI: −0.30 to −0.01).

LTPA trajectories across adulthood or current participation in different PA modalities at age 61 were not statistically significantly associated with blood glucose, systolic, or diastolic blood pressure.

## DISCUSSION

We aimed to identify LTPA trajectories across adulthood and to investigate the associations of LTPA trajectories and current participation in different PA modalities at the beginning of late adulthood, at age 61, with MetS and its components. We identified three LTPA trajectories over 34 yr, from age 27 to 61 yr. Those with consistently inactive and increasingly active LTPA trajectories over the follow-up had a higher risk of MetS, higher waist circumference, and dyslipidemia at 61 yr of age compared with those who remained consistently active. However, accounting for engagement in different PA modalities at age 61 attenuated these associations. Therefore, our results suggest that low LTPA across the adult lifespan could increase the risk of MetS and associate unfavorably with its components later in life, although PA participation, especially participation in muscle-strengthening activities and active commuting, at the beginning of late adulthood may mitigate the risk.

We identified relatively stable active and inactive LTPA trajectories, as well as an increasing LTPA trajectory from age 27 to 61. Interestingly, declining LTPA trajectories did not emerge in the present study sample. One explanation may be that the LTPA question was slightly broadened between the first two measurement waves to also capture incidental exercise. Only a very few studies have investigated LTPA trajectories for over three decades spanning from young adulthood to the beginning of late adulthood (22), such as in the present study. However, our results are in line with the existing literature suggesting that stable LTPA trajectories are more common compared with increasing or declining trajectories across the adult lifespan (22,45–47). Somewhat surprisingly, consistently active participants formed the largest group in the present study, followed by increasingly active. While consistently active participants reported on average exercising two to five times per week from age 27 to 61, increasingly active participants reached this level from age 42 to 61. Only one-fifth of the participants belonged to the consistently inactive group, exercising on average once a week or less frequently across adulthood, while low-active and inactive LTPA trajectories are typically most common in adulthood (47). In the increasingly active individuals, the LTPA level increase occurred between the ages of 27 and 42. It is worth noting that the broadening of the LTPA question occurred between these two measurement waves, which may explain the increase at least in some participants. Our finding, however, aligns with the findings of Berntzen et al, who found that changes in LTPA typically occurred between the ages of 30 and 40 in a larger population-based cohort of Finnish adults (22). In the future, it is important to investigate which factors influence changes in LTPA behavior in this age phase.

The findings of the present study indicate that maintaining a consistently active LTPA trajectory across adulthood is associated with a reduced risk of MetS at the beginning of late adulthood compared with those who have either consistently inactive or increasingly active LTPA trajectories. It is noteworthy that when participation in different PA modalities at age 61 was accounted for, only the elevated risk in the consistently inactive group remained statistically significant. To our knowledge, there are no previous studies investigating the mutual associations of LTPA across adulthood and current participation in different PA modalities with the risk of MetS in late adulthood. However, our findings support previous studies, which have consistently shown that physical inactivity is a significant risk factor for MetS. For instance, a systematic review and meta-analysis demonstrated that individuals with low levels of PA had a higher prevalence of MetS (48), while other studies have found that both persistent and increasing PA over time can reduce the risk of MetS (49,50). Our findings reinforce the importance of maintaining an active lifestyle throughout adulthood to mitigate the risk of MetS but also highlight that initiating LTPA in adulthood may support metabolic health in late adulthood when the metabolic risk typically increases (51).

When examining individual risk factors, our results, which showed that consistently inactive and increasingly active participants had higher waist circumference, lower HDL, and higher triglyceride levels compared with consistently active, support existing evidence. For example, the study by Martinez-Gomez et al. (52) highlighted that individuals with low PA levels had higher waist circumference and a higher risk of obesity and dyslipidemia. Zajac-Gawlak and colleagues (53), in turn, found that maintaining high daily step counts and increasing daily steps over a 7-yr follow-up were related to improved lipid profiles in women aged 60 yr and older, whereas no association was found between step counts and waist circumference (53). It is noteworthy that when current participation in muscle-strengthening, vigorous, commuting, and occupational PA was accounted for in the present analysis, LTPA trajectories were no longer statistically significantly associated with the individual risk factors. It may be that current PA may be more strongly associated with the current level of each risk factor, although the influence of LTPA across adulthood is reflected in the overall metabolic risk, that is, the presence of MetS. The lack of statistical significance may also partly be explained by the relatively small sample size, leading to a lack of statistical power.

It is noteworthy that not engaging in weekly muscle-strengthening activities was associated with higher waist circumference and less beneficial lipid profiles, while not commuting to work was associated with lower HDL. That is, engaging in these activities later in adulthood may, to some extent, mitigate the risks of low LTPA across the adult lifespan. Our findings highlight that participation in muscle-strengthening exercises, in addition to aerobic-type activities, is important in reducing the risk of metabolic dysfunction. Our results are supported by previous studies suggesting that resistance training may reduce adiposity and improve lipid profile in apparently healthy adults and those with metabolic conditions (54). Previous studies also have shown that participation in muscle-strengthening exercise is related to a lower risk of MetS (49,55) and may benefit metabolic health as a stand-alone intervention in individuals with overweight or obesity or metabolic disease (56). It is worth noting that approximately 40% of the age-cohort representative participants in the present study had BMI ≥30 kg/m^2^, which is a general criterion for obesity.

Cardiometabolic diseases, such as coronary heart disease and type 2 diabetes, remain a leading cause of morbidity and mortality (57). From a public health perspective, our findings underscore the critical need for promoting PA across the lifespan. Public health initiatives should focus on encouraging consistent PA from a young age across adulthood but also provide support for increasing activity levels at any phase of adulthood. This approach could help reduce the prevalence of MetS and its associated health complications, such as cardiovascular diseases, type 2 diabetes, vascular dementia, and musculoskeletal disorders (7,8,11), ultimately improving population health outcomes. Additionally, increasing LTPA across life from any baseline PA level reduces the overall mortality risk as well as mortality of cardiovascular diseases (58). While the greatest health benefits are seen when low-active individuals add at least some PA to their daily routine, the promotion of regular PA is essential at all phases of adult life, and targeting individuals who remain continuously physically inactive (i.e., low PA trajectories) would provide the largest returns (52,59). Our results also indicate that engaging in muscle-strengthening PA may be of importance for cardiometabolic health at the beginning of late adulthood, despite LTPA levels across adulthood.

This study has several limitations. First, the study sample was relatively small for the present purposes. This fact may have limited our capability to detect a greater number and more varied LTPA trajectories, as well as to investigate potential differences in trajectories between men and women. However, according to the systematic review by Lounassalo et al. (47), three to five distinct LTPA trajectories have typically been found in various populations. Second, the present study relies on self-reported PA data, which may be subject to recall bias and inaccuracies. We used a single-item global question on the weekly frequency of LTPA, which has not been validated against a golden standard measure and does not provide information about the total volume or specific types of LTPA. However, other similar one- and two-item questionnaires have shown relatively good reliability and validity, and they have been deemed useful in identifying PA profiles (61). Since PA was not the initial focus of the JYLS study, a global questionnaire was chosen to describe participants’ overall PA level. In addition, the phrasing of the question slightly changed from the first data collection wave at age 27 to the following data collections at the ages of 42, 50, and 61. During the later three waves, the question covered incidental exercise, which may, for example, include walking for errands, commuting to work, household chores, and yard work, in addition to training and sport participation. This may have led to categorizing some participants as increasingly active despite unchanged LTPA behavior. Furthermore, we had to recategorize the 7-point scale question used from age 42 to 61 to match the 5-point scale question at age 27. That is, the category 4, to which consistently active individuals belonged through their adulthood and increasingly active from age 42 to 61, was very broad (exercise 2–5 times per week). Moreover, due to the focus on LTPA frequency and lack of information on duration and intensity, it is not possible to distinguish between moderately and highly active individuals. These limitations highlight the importance of also exploring participation in various PA modalities, for which data were unfortunately available only from the latest data collection.

An important limitation is that we could not account for several potentially confounding factors, such as dietary habits and genetic predispositions, which could influence the observed associations. Furthermore, since we only investigated MetS at age 61, we cannot exclude the opportunity for reverse causality. That is, it is possible that individuals with better metabolic health earlier in life may have been able to be physically active through adulthood. Future research is needed in larger study samples having as intensively collected data as in the present study and including device-based LTPA assessment in addition to self-reported PA assessment methods to confirm the findings from the present study.

Despite the limitations, this study also has several strengths. Only a very few studies have thus far investigated LTPA prospectively for over 3 decades. The longitudinal design of the present study, spanning over 34 yr, provides robust data on LTPA trajectories and their long-term health impacts. This study also benefits from a comprehensive assessment of multiple MetS components, offering a detailed understanding of the relationship between LTPA and metabolic health. Finally, in addition to inspecting the general LTPA trajectories across adulthood, we included measures of participation in different PA modalities at the beginning of late adulthood, highlighting the association between muscle-strengthening PA and MetS. Finally, the generalizability of the present findings is relatively good in a comparable population, since there was no initial dropout in the recruitment, and the participants of the latest data collection wave, TRAILS, are still relatively well representative of the Finnish age cohort born in 1959 (30).

## CONCLUSIONS

In conclusion, our study highlights the significant role of both long-term PA trajectories and current participation in PA on the risk of MetS and its components. Individuals who were inactive in young adulthood and thereafter either remained inactive or increased their LTPA in midlife had higher risks of MetS, high waist circumference, and dyslipidemia at 61 yr of age compared with those who were consistently active across adulthood. However, participation in different PA modalities, especially muscle-strengthening activities, at the beginning of late adulthood attenuated these risks. These findings emphasize both the importance of promoting and sustaining PA throughout life to improve metabolic health and reduce the burden of MetS in the aging population.

The most recent JYLS data collection, called TRAILS, was funded by the Academy of Finland under Grant 323541 to K. K. The work of T. S. was supported by a personal grant from the Juho Vainio Foundation awarded in 2024. The work of J. A. and E. R. was supported by personal grants from the Finnish Cultural Foundation awarded in 2022 and 2023, respectively. The funders did not have any role in the preparation of the present manuscript. No conflicts of interest were disclosed. All authors have made substantial contributions to the design of the study and the manuscript. T. S. and E. A. H. were responsible for conceptualization and preparing the original manuscript draft. T. S. analyzed the data. K. K., E. R., J. A., T. K., and M. L. K. reviewed and edited the manuscript. K. K. was responsible for funding acquisition and project administration. All authors were responsible for data curation and investigation. All authors revised the manuscript critically for important intellectual content and approved the final version to be published. All authors agreed to be accountable for all aspects of the work in ensuring that questions related to the accuracy or integrity of any part of the work are appropriately investigated and resolved. The study was conducted in agreement with the Helsinki Declaration. The study protocols for data collections conducted in 2001 and 2009 were approved by the Ethical Committee of the Central Finland Health District (nos. 42/2000, 10E/2008, respectively). The study protocol for the data collection in 2020–2021 was approved by the Ethical Committee of the University of Jyväskylä (December 13, 2019). The participants signed an informed consent before participation in each data collection wave. The dataset analyzed during the current study is not publicly available due to the sensitivity of the data and privacy of the participants. The data analyses that support the findings of the present article are available from the corresponding author upon reasonable request. Pseudonymized dataset is available to external collaborators upon agreement on the terms of data use and publication of results (30). To request the data, contact the Principal Investigator of the JYLS/TRAILS study, Dr. Katja Kokko (katja.r.kokko@jyu.fi). The results of the study are presented clearly, honestly, and without fabrication, falsification, or inappropriate data manipulation. The results of the present study do not constitute endorsement by the American College of Sports Medicine.

## Supplementary Material
