# Supplementary material for: Leisure-Time Physical Activity Trajectories across Adulthood and Cardiometabolic Risk at the Beginning of Late Adulthood: A Prospective Cohort Study
Source: Med Sci Sports Exerc. 2025 Oct 27;58(3):493–505. doi: 10.1249/MSS.0000000000003883 (PMC12863631; doi:10.1249/MSS.0000000000003883)
Supplement: Supplementary file 1 [file msse-58-493-s001.pdf]

Supplementary Table 1. Participant characteristics at age 50 in the analytical sample compared to the participants excluded from the analysis due to missing data.

	**Analytical sample (N=159)**	**Excluded participants (N=47)**	**P [1]**
Gender, female, n (%)	83 (52)	24 (51)	0.891
BMI, kg/m^2, mean ± SD [2]	27.3 ± 4.7	27.1 ± 5.2	0.871
Good/very good self-reported health, n (%) [3]	109 (70)	31 (69)	0.853
Occupational status, n (%)			0.797
Upper white collar	50 (31)	14 (30)	
Lower white-collar	68 (43)	22 (48)	
Blue collar	41 (26)	10 (22)	
LTPA trajectory			0.937
Consistently active	67 (42)	20 (43)	
Increasingly active	58 (36)	16 (34)	
Consistently inactive	34 (21)	11 (23)	

Note

[1] P values for categorical values from Chi squared tests and for continuous variables from independent samples t-tests.

[2] Missing N=9 in the analytical sample, N=11 in the excluded sample.

[3] Missing N=4 in the analytical sample, N=2 in the excluded sample.

Abbreviations: BMI = body mass index; SD = standard deviation; LTPA = leisure-time physical activity.
